# Reconstitution of high-grade serous ovarian carcinoma from primary fallopian tube secretory epithelial cells

**DOI:** 10.18632/oncotarget.23035

**Published:** 2017-12-08

**Authors:** Kohei Nakamura, Kentaro Nakayama, Noriyoshi Ishikawa, Masako Ishikawa, Razia Sultana, Tohru Kiyono, Satoru Kyo

**Affiliations:** ^1^ Department of Obstetrics and Gynecology, Shimane University School of Medicine, 6938501, Izumo, Japan; ^2^ Department of Organ Pathology, Shimane University School of Medicine, 6938501, Izumo, Japan; ^3^ Division of Carcinogenesis and Cancer Prevention, National Cancer Center Research Institute, 1040045, Tsukiji, Chuo-ku, Japan

**Keywords:** carcinogenesis, high-grade serous ovarian carcinoma, fallopian tube, RAS/PI3K pathway, c-Myc

## Abstract

Fallopian tube secretory epithelial cells (FTSECs) have been suggested to be the source of high-grade serous ovarian carcinoma (HGSOC). Although several genetic alterations are known to be involved in HGSOC development, the minimal requirements remain unclear. We aimed to identify oncogenic mutations indispensable for HGSOC development in a stepwise model, using immortalized FTSECs. FTSECs were isolated from clinical samples and immortalized by overexpression of cyclin D1, CDK4^R24C^, and *hTERT*. Oncogenic mutations in the p53, c-Myc, and RAS/PI3K pathways were mimicked by lentiviral transduction. We found two distinct patterns of gene alteration essential for HGSOC development: p53/KRAS/AKT and p53/KRAS/c-Myc. Dominant-negative p53, alone or combined with oncogenic *KRAS* (*KRASV12*), constitutively active AKT (CA-AKT), and c-Myc, did not induce tumorigenesis in immortalized cells; however, overexpression of CA-AKT or c-Myc, along with dominant-negative p53 and *KRASV12*, conferred tumorigenic potential. Transformed FTSECs formed tumors in nude mice that were grossly, histologically, and immunohistochemically similar to human HGSOCs. Interestingly, mice harboring tumors with c-Myc amplifications displayed extensive metastases, consistent with the increased dissemination in their human counterparts. Thus, aberrant p53/*KRASV12*/c-Myc or p53/*KRASV12*/PI3K-AKT signaling was the minimum requirement for FTSEC carcinogenesis. The model based on this evidence could shed light on the early stages of HGSOC development.

## INTRODUCTION

Epithelial ovarian cancer (EOC) is a heterogeneous group of neoplastic diseases that comprise the most lethal gynecologic malignancy worldwide [1]. Several cellular origins have been reported for EOCs, including epithelial cells of the fallopian tube fimbriae, ovarian fimbriae, ovarian surface epithelium, inclusion cysts, peritoneal mesothelium, or endometriotic tissue. The hypothesis of fallopian tube fimbriae-derived high-grade serous ovarian carcinomas (HGSOCs), particularly with respect to fallopian tubal secretory epithelial cells (FTSECs), has been attracting increasing attention in recent years [2–5].

Serous carcinoma precursor lesions often arise in the fallopian fimbriae proximal to the ovary as a result of p53 inactivation, termed “p53 signature” lesions [6–9]. Many p53 signatures share identical somatic *TP53* mutations with coexisting serous tubal intraepithelial carcinomas and HGSOCs, suggesting a common origin for all three entities [8]. Furthermore, p53 signatures are rarely observed in ovarian surface epithelial (OSE) cells [10]. Collectively, these observations support the hypothesis that HGSOCs arise from the fallopian tube fimbriae.

Comprehensive genetic analyses have been performed in HGSOCs [11]; however, the events necessary for carcinogenesis remain unclear. In this study, we aimed to generate a model to investigate the early stages of HGSOC formation by establishing immortal FTSECs and simulating the genetic lesions found in clinical cases of HGSOC by exogenous expression of various oncogenic genes. Cells with one or more of these lesions were examined for tumorigenicity, both *in vitro* and *in vivo*.

Immortal cell lines derived from normal human tissue are essential tools for characterizing the molecular pathways in ovarian carcinogenesis. Karst et al. established an immortal cell line from human fallopian tube tissue [12]; however, they found that the growth rate and morphology of the immortalized cells changed over time, and long-term culturing eventually caused the accumulation of genetic defects. We had previously reported that co-expression of cyclin D1 and mutant cyclin-dependent kinase 4 (CDK4^R24C^) was required to overcome the premature senescence of secretory epithelial cells, and that these genes, combined with telomerase (*hTERT*) expression, were sufficient for cell immortalization [13]. Importantly, this method does not require transformation with viral oncogenes such as SV40 that may be associated with carcinogenesis.

HGSOCs are aggressive and often presented in an advanced stage. As such, these cancers are associated with poor prognoses. Previous analyses had identified several genetic abnormalities in oncogenes and tumor suppressors involved in HGSOC tumorigenesis. *TP53* mutations were the most prevalent, and were observed in approximately 96% of cases; activating mutations of the retinoblastoma (Rb) pathway were also common [11]. The RAS/PI3K pathway is the key signaling pathway in HGSOC and is deregulated in 45% of tumors [11]. The RAS/PI3K pathway can be divided into its RAS/ERK and PI3K/AKT constituents, which are independently affected by genetic factors such as *KRAS* and *BRAF* lesions or *PTEN* and *PIK3CA* mutations, respectively [11]. Despite accumulating evidence regarding the prevalence of these molecular alterations, the specific combination of genetic mutations required for HGSOC development has not been well investigated. Furthermore, c-Myc, an oncogene encoding a transcription factor that plays a fundamental role in cell proliferation, growth, differentiation, and apoptosis, is overexpressed in many HGSOCs [11]; however, the mechanism by which this protein contributes to HGSOC development remains unknown. These oncogenetic observations motivated us to investigate the minimal genetic requirements necessary for HGSOC tumor formation.

In this study, our first challenge was to establish a method for immortalizing FTSECs while retaining their normal phenotype. Our second challenge was to identify the minimal number and specific combination of mutations in samples from patients with HGSOC that would be sufficient for malignant transformation of the immortalized FTSECs. We thus aimed to generate an *in vitro* stepwise model of carcinogenesis. In the future, this model will be useful for dissecting the molecular mechanisms of HGSOC development, as well as for developing oncogenic pathway-specific inhibitors.

## RESULTS

### Establishment of immortalized FTSECs

**Figure 1:**
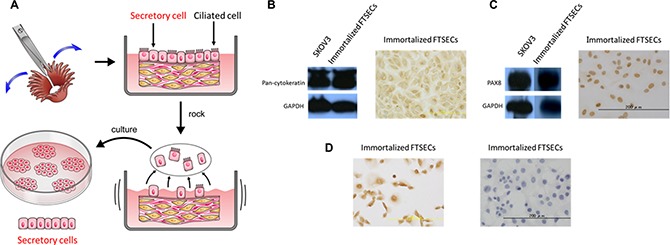
Fallopian tube secretory epithelial cell (FTSEC) isolation and characterization (**A**) Tissue acquisition and purification, including dissection (upper-left panel), culturing in DMEM with 5% FBS and 1% PenStrep (upper-right panel), epithelial cell disruption (lower-right panel), and acquisition of adherent secretory cells (lower-left panel). (**B**) Western blot (left panel) and immunocytochemical (right panel) analyses of Pan-cytokeratin expression in immortalized FTSECs. (**C**) Western blot (left panel) and immunocytochemical (right panel) analyses of PAX8 expression in immortalized FTSECs. (**D**) Immunocytochemical analysis of Bcl2 (left panel) and FOXJ1 (right panel) expression in immortalized FTSECs.

Fallopian tube tissues were collected from two patients with leiomyoma who were undergoing total laparoscopic hysterectomy and bilateral salpingo-oophorectomy. FTSECs were isolated (Figure 1A). Kiyono et al. had previously reported that co-expression of activated CDK4 (CDK4^R24C^), cyclin D1, and hTERT is sufficient to immortalize epithelial cells [12]. We therefore introduced these factors into primary FTSECs using recombinant lentiviruses. Although no differences were observed in the morphology between the primary and immortalized FTSECs (Supplementary Figure 1A, 1B), the immortalized cells, in contrast to the parental cells, showed accelerated growth with no signs of slowing over 180 days of culturing (Supplementary Figure 1C). Subsequently, western blotting and immunostaining for the epithelial marker, cytokeratin, and the Müllerian and secretory cell marker, PAX8, were performed to assess stromal cell contamination. Immunostaining for BCL2 and FOXJ1 was performed to detect secretory and ciliated cells, respectively. As expected, all adherent cells expressed cytokeratin, PAX8, and BCL2, but not FOXJ1 (Figure 1B–1D). Furthermore, the absence of cell colonies in soft-agar assays and xenograft tumors, even 9 months after engraftment (data not shown), confirmed that the immortalized FTSECs did not acquire a transformed phenotype. Together, these findings demonstrated that our immortalized FTSECs were pure and non-transformed, and did not dedifferentiate upon prolonged cultivation.

### Identification of key oncogenetic alterations in human HGSOC samples

Although comprehensive genetic analyses of HGSOCs have been performed [11], little information is available regarding the Japanese population. Therefore, based on TCGA data, we investigated the genetic alterations in HGSOC samples from Japanese patients. Thirty-four patients with HGSOC receiving treatment at the Shimane University Hospital from January 2011 to December 2015 were enrolled in our study. The median age at diagnosis was 63 years (range, 41–80 years). Three (8.9%) and 31 (91.1%) patients were classified as having FIGO Stage I/II and Stage III/IV disease, respectively. Nine patients (26.5%) were classified as having grade 2 tumors, whereas 25 patients (73.5%) were classified as grade 3.

All 34 cases were analyzed for mutations at key genomic loci, as well as for pathway activation status. Notably, all HGSOCs (100%) exhibited exon mutations in *TP53.* Additionally, 14 tumors (41.2%) harbored *PTEN* mutations, including 8 with point mutations and 6 with frameshift mutations; two (5.9%) harbored a missense mutation in *PIK3CA* exon 20, two (5.9%) had *KRAS* mutations, and one (2.9%) had a *BRAF* mutation. Moreover, FISH and CISH analyses detected *KRAS* and *PIK3CA* amplification in three (8.8%) and 11 (32.4%) patients, respectively, whereas c-Myc amplification was detected in 13 (38.2%) patients. These results were consistent with the data in the TCGA database.

**Figure 2:**
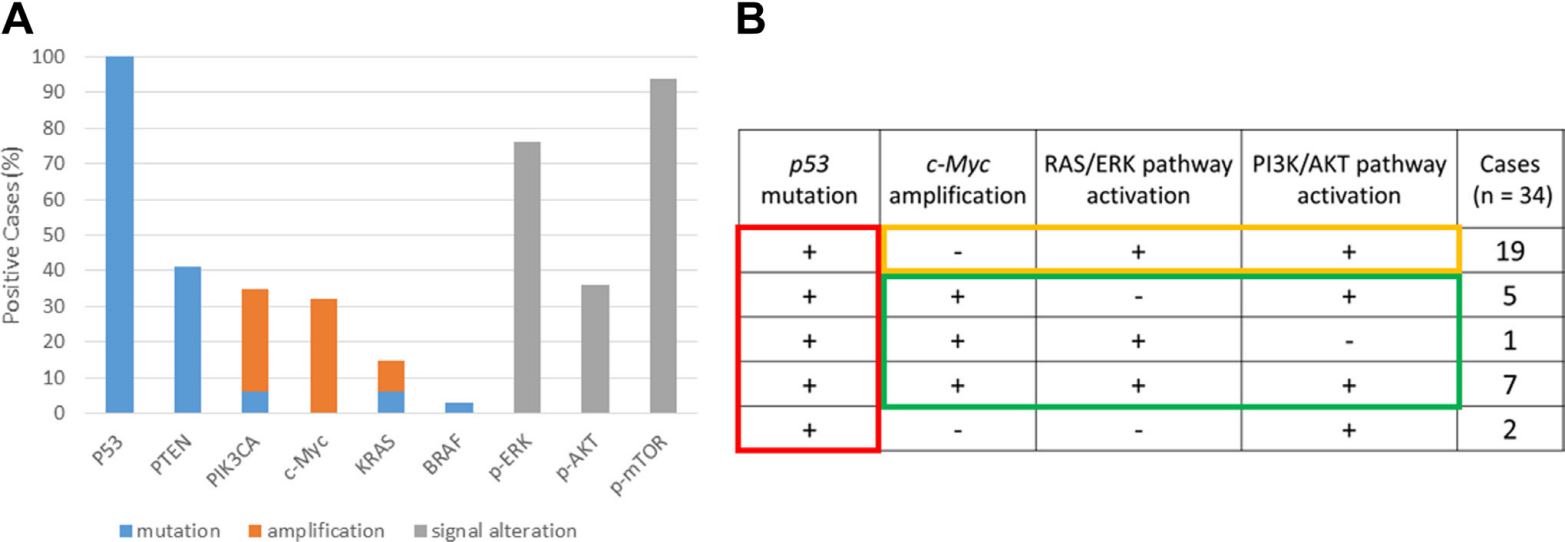
Mutation, amplification, and pathway activation status of tumor samples from patients with high-grade serous ovarian carcinoma (HGSOC) (**A**) Status of P53, PTEN, PIK3CA, KRAS, and BRAF mutations; PIK3CA, c-Myc, and KRAS amplification; and RAS/ERK and PI3K/AKT pathway activation in HGSOC tumors. (**B**) Analysis of p53 mutation, c-Myc amplification, and activation of the RAS-ERK and PI3/AKT pathways in 34 patients with HGSOC.

Pathway activation studies found phosphorylated (p-)MAPK, p-AKT, and p-mTOR in 26 (76.5%), 13 (38.2%), and 32 (94.1%) of the tumors, respectively, as determined by IHC (Figure 2A). Notably, p-MAPK and p-AKT localized to both the cytoplasm and nucleus, whereas p-mTOR was retained only within the cytoplasm. Images of positive and negative controls are presented in Supplementary Figure 2. The association between MAPK/AKT/mTOR activation and KRAS/PTEN/PIK3CA mutation status was assessed using the X^2^ test, which revealed a significant correlation between p-AKT/p-mTOR and PTEN/PIK3CA (*P* = 0.001; data not shown), and between p-MAPK and KRAS (*P* = 0.03; data not shown).

We subsequently focused on the status of the *TP53* mutation, c-Myc amplification, RAS/ERK pathway, and PI3K/AKT pathway. For the purpose of this analysis, RAS/ERK pathway activation was defined as p-MAPK positive, and PI3K/AKT pathway activation as p-AKT- or p-mTOR-positive (Figure 2A). Interestingly, among the 21 cases lacking c-Myc amplification, 15 (88%) displayed constitutive activation of both RAS/ERK and PI3K/AKT, whereas only one pathway needed to be activated in the presence of c-Myc amplification (*n* = 13; Figure 2B). Based on these findings, we hypothesized that *TP53* inactivation is a predisposing step in HGSOC initiation, and that any two of RAS/ERK activation, PI3K/AKT activation, or c-Myc amplification comprise candidate key elements for HGSOC development.

### Putative stepwise model of HGSOC development

**Figure 3:**
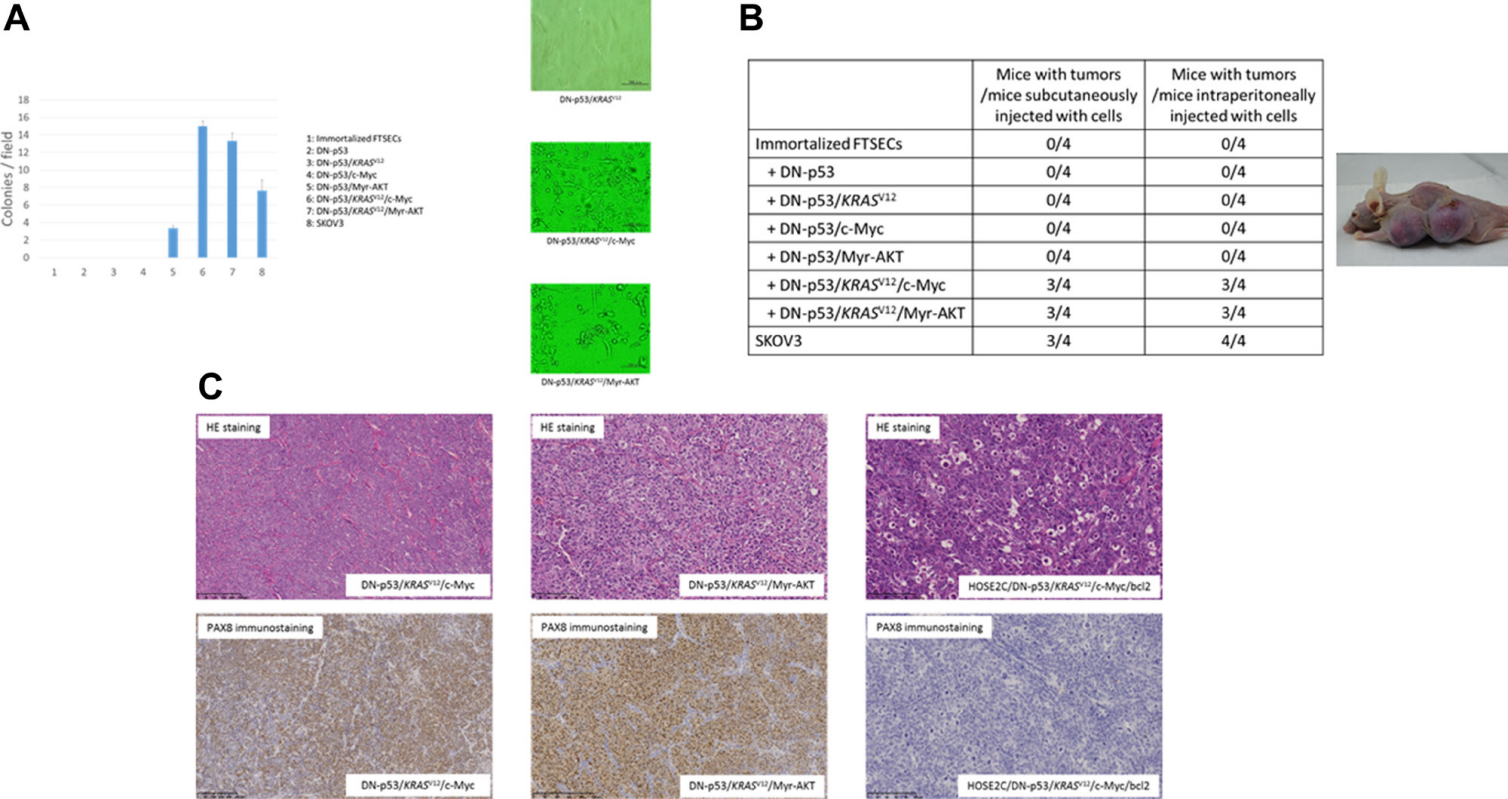
Anchorage-independent growth in soft agar and analysis of transformed phenotypes in mouse xenografts (**A**) Growth of (1) immortalized FTSECs; immortalized FTSECs expressing (2) DN-p53, (3) DN-p53/*KRAS*^V12^, (4) DN-p53/c-Myc, (5) DN-p53/Myr-AKT, (6) DN-p53/*KRAS*^V12^/c-Myc, (7) and DN-p53/*KRAS*^V12^/Myr-AKT; and (8) SKOV3 serous ovarian carcinoma cells (positive control) was evaluated in soft agar (left panel), and representative colony images (DN-p53/*KRAS*^V12^: right-upper panel, DN-p53/*KRAS*^V12^/c-Myc: right-middle panel, DN-p53/*KRAS*^V12^/Myr-AKT: right-lower panel). (**B**) Tumor formation assay using fallopian tube secretory epithelial cells (FTSECs) transfected with different genetic elements. SKOV3 ovarian carcinoma cells were injected as a positive control (right panel). Subcutaneous injection of cells with DN-p53/*KRAS*^V12^/c-Myc or DN-p53/*KRAS*^V12^/Myr-AKT expression resulted in tumor formation in nude mice (left panel). (**C**) Hematoxylin and eosin (HE) staining and PAX8 immunostaining of tumors formed from FTSECs expressing DN-p53/*KRAS*^V12^/c-Myc (left panels) or DN-p53/*KRAS*^V12^/Myr-AKT (middle panels) or from immortalized ovarian surface epithelial cells transfected with DN-p53, *KRAS*^V12^, c-Myc, and BCL2 (right panels).

We next sought to establish a stepwise model of *in vitro* HGSOC development using immortalized FTSECs. Because *TP53* mutations were found in 100% of the HGSOCs analyzed, we hypothesized that this was an initiating event necessary for carcinogenesis and simulated it in the immortalized FTSECs by overexpressing DN-p53. RAS/ERK or PI3K/AKT pathway activation was then induced in FTSEC-DN-p53 cells by transfection with vectors expressing *KRAS*^V12^ or Myr-AKT, respectively. The transformed cell phenotypes were subsequently evaluated by soft agar colony formation assays and xenograft tumor assays in nude mice. Notably, although neither non-transfected FTSECs nor their DN-p53, DN-p53/*KRAS*^V12^, or DN-p53/c-Myc derivatives formed colonies in soft agar or tumors in mice, FTSECs expressing both DN-p53 and Myr-AKT formed colonies, but were unable to generate tumors (Figure 3A, 3B). Conversely, the DN-p53/*KRAS*^V12^/Myr-AKT and DN-p53/*KRAS*^V12^/c-Myc cell lines formed significantly more colonies than those expressing DN-p53 and Myr-AKT (*P* < 0.001) and formed tumors in mice (4/4, 100%). The transfection efficiency and pathway activation were assessed in each generated cell line by western blot analysis, as shown in Supplementary Figure 3. Additionally, expression of p53/KRASV12/c-Myc or DN-p53/KRASV12 /Myr-AKT in the xenograft tumors was validated by immunohistochemistry (Supplementary Figure 4).

**Figure 4:**
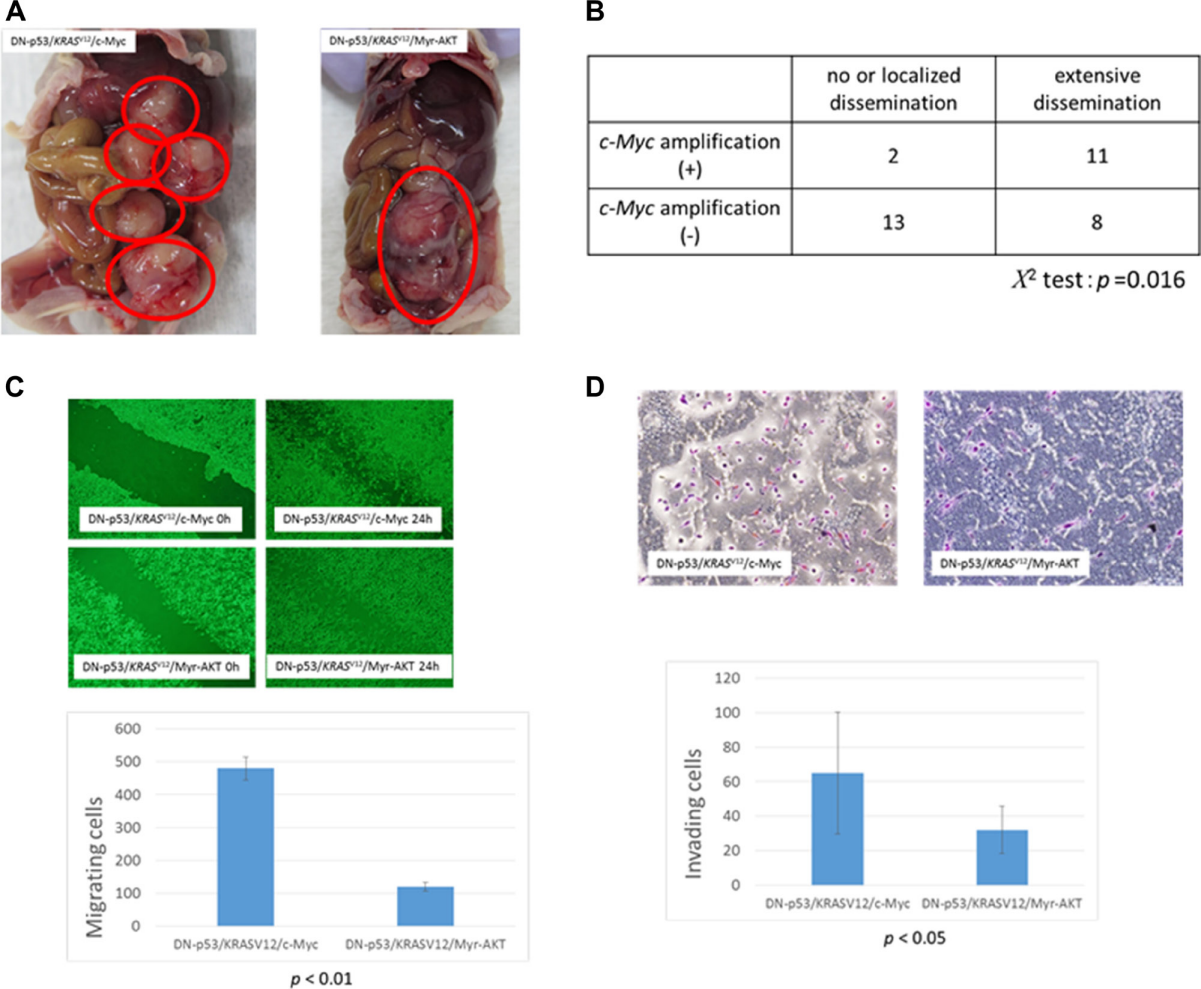
Association between c-Myc amplification and metastasis in high-grade serous ovarian carcinoma (HGSOC) (**A**) Tumors formed in nude mice after intraperitoneal injection of fallopian tube secretory epithelial cells (FTSECs) expressing DN-p53/*KRAS*^V12^/c-Myc (left panel) or DN-p53/*KRAS*^V12^/Myr-AKT (right panel). (**B**) Correlation between c-Myc amplification and degree of dissemination in patients with HGSOC. (**C**) Migration of FTSECs expressing DN-p53/*KRAS*^V12^/c-Myc and DN-p53/*KRAS*^V12^/Myr-AKT in wound healing assays (0 h, left panels; 24 h, right panels). (**D**) Matrigel invasion assay analysis of FTSECs expressing DN-p53/*KRAS*^V12^/c-Myc (upper panel) or DN-p53/*KRAS*^V12^/Myr-AKT (lower panel). Error bars indicate standard deviation.

Histological examination of the xenograft tumors derived from the DN-p53/*KRAS*^V12^/c-Myc and DN-p53/*KRAS*^V12^/Myr-AKT cell lines showed poorly differentiated carcinomas with high nucleus-to-cytoplasm (N/C) ratios that grew as solid tumor nests. Moreover, the tumors exhibited diffuse expression of PAX8, WT1, and CK7, but no CK20 expression, suggesting that they were high-grade serous carcinomas with Müllerian origin (Figure 3C). No pathological differences were observed between the subcutaneous and intraperitoneal tumors. Ovarian surface epithelial cells were considered the origin of HGSOCs for many years, and were previously utilized in a carcinogenetic model with p53 inactivation, oncogenic *KRAS*^V12^, and c-Myc and Bcl-2 overexpression [21]. However, these tumors are highly anaplastic, with narrow mitotic poles and apoptotic bodies, and lack PAX8 expression (Figure 3C). Moreover, they grow as solid sheets of loosely associated epithelial cells in mice, and are thus more representative of undifferentiated carcinomas than HGSOCs [21]. Interestingly, FTSEC DN-p53/*KRAS*^V12^/c-Myc intraperitoneal tumors were highly disseminated in the peritoneal organs, including the liver, diaphragm, and digestive tract, whereas DN-p53/*KRAS*^V12^/Myr-AKT cells formed solid tumors without dissemination (Figure 4A). These findings suggest that *c-Myc* mutations played an important role in cancer dissemination.

### Correlation between c-Myc amplification and degree of dissemination in HGSOC

To investigate the role of *c-Myc* in HGSOC dissemination, we analyzed the association between *c-Myc* amplification and metastasis. Patients were classified as having extensive dissemination or no/localized dissemination, as summarized in Figure 4B. Among the 13 patients with *c-Myc* amplification, 11 (85%) exhibited extensive dissemination, whereas only 8 (38%) had no amplification. Furthermore, correlation analysis showed that *c-Myc* amplification was significantly associated with extensive dissemination in patients with HGSOCs (*P* = 0.016, Figure 4B). We performed analyses to compare myc amplification and clinical stage, and other clinicopathological factors, but no significant differences were observed.

We also assessed differences in the motility of DN-p53/*KRAS*^V12^/c-Myc and DN-p53/*KRAS*^V12^/Myr-AKT cells via wound healing and Matrigel invasion assays. Notably, DN-p53/*KRAS*^V12^/c-Myc FTSECs showed a ~200% increase in motility compared to DN-p53/*KRAS*^V12^/Myr-AKT cells in both assays (*P* < 0.01, Figure 4C, 4D), suggesting that c-Myc amplification might lead to extensive dissemination via increased cell motility and invasion. However, it should be noted that differences in proliferation between DN-p53/KRASV12/c-Myc and DN-p53/KRASV12/Myr-AKT may have confounded the cell migration and invasion analyses.

Lastly, the participation of c-Myc in the proliferation and motility of DN-p53/*KRAS*^V12^/c-Myc FTSECs was assessed by treating the cells with the c-Myc inhibitor 10058-F4 (Supplementary Figure 5A). Interestingly, although 10058-F4 treatment hindered cell proliferation (Supplementary Figure 5B), no profound effects on cell migration or invasion were observed (Supplementary Figure 5C, 5D).

## DISCUSSION

The most important achievement of the present study was the successful reconstitution of HGSOCs *in vitro*, as evidenced by gross, histological, and immunohistochemical analyses, using human fimbrial cells. The keys to the success of this reconstitution were the high purity of the FTSEC cells, which was achieved by gatefolding the fimbriae and rocking them gently in culture medium to prevent stromal cell contamination, and the non-transformed phenotype of the immortalized cells despite long-term culture. Given the uncertainty regarding the origin of this disease, a reliable model such as ours will provide various directions not only for studies of carcinogenesis, but also for identifying the molecular targets of HGSOCs.

Karst et al. previously established an *in vitro* HGSOC model [12], which had a considerable impact on the field of gynecology. In their study, FTSECs were immortalized with *hTERT*, *p53*-shRNA, and *CDK4*; however, the growth rate and morphology of the immortalized FTSECs likely changed over time [12]. Specifically, because *TP53* loss of function is associated with various genetic rearrangements, the immortalized characteristics of these cells can change during long-term cultivation. Furthermore, they utilized mutant *HRAS* and *c-Myc* overexpression to create a model of fallopian tube-derived HGSOC; however, *HRAS* mutations are rarely found in samples from patients with HGSOC [22]. Therefore, we established a new, more clinically relevant model of carcinogenesis by immortalizing FTSECs through introduction of cyclin D1, CDK4^R24C^, and *hTERT*, and thus generated an HGSOC model harboring only *TP53*, *KRAS*, *AKT*, and c-Myc alterations, which are frequently observed in clinical situations. Sasaki et al. previously established a carcinogenetic model comprising OSE cells immortalized by p53 inactivation, oncogenic *KRAS*^V12^, and c-Myc and Bcl-2 overexpression [21]. However, tumor growth in mice revealed undifferentiated carcinomas devoid of PAX8 expression, which were not similar to human HGSOCs (Figure 3C) [21]. These data therefore support our hypothesis that FTSECs are the origin of HGSOCs. In our analysis, the ratio of PTEN mutation was much higher than that in previous studies. However, high frequencies of heterogeneous PTEN mutation were also seen in HGSOCs, and 61% of HGSOC cases in a previous study had PTEN mutations [23]. We consider that these differences might arise from sample heterogeneity or racial differences.

Our *in vitro* data showed that *TP53* inhibition, along with *KRAS*^V12^, Myr-AKT, or c-Myc overexpression, failed to induce the transformation of immortalized FTSECs, whereas either c-Myc or Myr-AKT overexpression in cells with *TP53* inactivation and *KRAS*^V12^ expression effectively conferred a transformed phenotype. These findings suggest that a limited set of genetic lesions, including Rb inactivation, telomerase activation, *TP53* inactivation, and either constitutive RAS/PI3K pathway activation by *KRAS*^V12^ or *Myr-AKT* expression or c-Myc overexpression, was sufficient to induce neoplastic transformation, and that widespread genomic changes were not always necessary for this process.

A recent study indicated that homologous recombination deficiency and gain/amplification of CCNE1 copy number occur early in tumor progression and precede centrosome amplification in HGSOCs [24]. However, homologous recombination deficiency and cyclin E1 amplification were not included in this model. We are now trying to establish a new model including these factors.

Cancer arises through the sequential accumulation of mutations in oncogenes and tumor suppressors. Previous studies had identified only two genetic mutations that were necessary for tumor initiation and progression in mice [22, 25, 26]. This discrepancy could be explained by differences in assay conditions (*in vitro* vs. *in vivo*) or between humans and mice. Recently, Vogelstein et al. concluded that three sequential mutations were required to drive human colon and lung tumorigenesis using a mathematical approach with genome-wide sequencing data [27, 28], which was similar to the findings obtained with our *in vitro* HGSOC model.

There appeared to be a clear dissociation of the role of *KRAS*^V12^ mutation *in vitro* and *in vivo*. Although the combination of Myr-AKT or c-Myc overexpression with *KRAS*^V12^ was sufficient to transform cells in the presence of DN-p53 cells, *KRAS*^V12^ overexpression alone failed to do so, indicating that a combination of mutations was necessary for transformation. In contrast, *KRAS*^V12^ mutations in HGSOCs were demonstrated to be infrequent (11%) in a previous study [11]. One possible explanation for this discrepancy is that *KRAS* is located upstream of RAS/ERK signaling, and that various downstream factors, apart from the *KRAS* mutation, might also be associated with carcinogenesis, including epigenetic or microRNA regulation of the signal pathway.

Anomalies within the c-Myc locus, such as gene amplification, are observed in 20% of HGSOCs [11]. Our *in vitro* model clearly demonstrated the role of c-Myc overexpression in the transformation of immortalized FTSECs, in combination with *TP53* inhibition and *KRAS*^V12^. The cooperative roles of RAS/ERK signaling with Myc in neoplastic transformation have also been demonstrated in other cell types, where RAS/ERK signaling promotes Myc protein stability via Ser 62 phosphorylation [29]. In fact, our study clearly showed that c-Myc overexpression in the absence of *KRAS*^V12^ was insufficient for transformation, whereas Myr-AKT overexpression transformed FTSECs in the presence of DN-p53 *KRAS*^V12^ without c-Myc overexpression, suggesting that the RAS/PI3K pathway is a promising target to suppress the tumorigenic phenotype of HGSOCs. Furthermore, we showed that c-Myc amplification was associated with the degree of dissemination and promoted cell proliferation *in vitro*, indicating that this pathophysiological mechanism might be responsible for the challenges associated with the resection of tumors harboring c-Myc amplification. Thus, c-Myc inhibitor therapy, in combination with debulking surgery, could potentially prolong the survival of these patients.

In summary, we identified two patterns of three genetic alterations (i.e., p53/*KRAS*^V12^/c-Myc and p53/*KRAS*^V12^/PI3K-AKT) that were essential for HGSOC development, and successfully established an *in vitro* step-wise model of carcinogenesis using immortalized FTSECs. This experimental model provides a foundation for future studies on early HGSOC development, and for identifying candidate targets for drug development and secreted biomarkers for early detection.

## MATERIALS AND METHODS

### FTSEC isolation

Human fallopian tube tissue samples were obtained from two patients (aged 46 and 64 years) who were undergoing total laparoscopic hysterectomy and bilateral salpingo-oophorectomy as treatment for uterine myoma. All methods were approved by the Institutional Research Ethics committee of Shimane University (Izumo, Japan), and written informed consent was obtained from each patient. Fresh fallopian tube fimbriae were collected in a 10-cm tissue culture dish, washed with sterile PBS, and then cut longitudinally using a scalpel and tweezers to maximize the exposure of the epithelial cells (Figure 1A, upper-left panel). Dissected fimbriae were transferred to a 25-cm^2^ dish containing 5 mL Dulbecco’s modified Eagle medium (DMEM, Cat No. D5796; Sigma-Aldrich, St. Louis, MO, USA) supplemented with 5% fetal bovine serum and 1% PenStrep, and rocked gently at room temperature for 48 h to dissociate the epithelial cells from the fimbriae (Figure 1A, lower-right panel). Caution was taken to avoid rocking the plates violently to prevent stromal cell contamination. The dissociated cells and fimbrial tissues were then cultured in a humidified 37 °C incubator with 5% CO_2_, with medium changes every 2–3 days. After 7 days, clusters of adherent secretory cells were ~30% confluent (Figure 1A, lower-left panel), at which point the fimbrial tissues were removed and the medium was replenished every 2–3 days until the cells reached 60–70% confluency. Ciliated epithelial cells can occasionally contaminate primary FTSEC cultures, but this does not present an issue because the cells cannot persist in 2D culture or may undergo transdifferentiation to the secretory phenotype when cultured *in vitro* [12].

### Viral vector construction and cell transduction

Lentiviral vector plasmids were constructed using the Gateway system (Invitrogen, Carlsbad, CA, USA). Briefly, the genes encoding hTERT, human cyclin D1, and inhibitor-resistant human CDK4 (CDK4^R24C^, generously provided by Dr. Hara) were first inserted into entry vectors, and then transferred into the CSII-CMV-RfA lentiviral destination vector (gifted by Dr. Miyoshi), generating CSII-CMV-hTERT, -cyclin D1, and -hCDK4R24C, respectively. Recombinant lentiviruses were produced with vesicular stomatitis virus G glycoprotein, as described previously [14]. Immortalized FTSECs were used in their original form without antibiotic selection. Genes encoding mutant *KRAS* (*KRAS*^V12^), constitutively active AKT1 (myristoylated human AKT1 [Myr-AKT1], a gift from Dr. Goto), c-Myc, and dominant-negative p53 (DN-p53) [15] were then cloned into lentiviral vector plasmids. These vectors, pCLXSN-*KRAS*^V12^, pCLMSCVpuro-Myr-AKT, pCLMSCVpuro-c-Myc, and pCLMSCVbsd-DN-p53, have been described previously [16]. Cells were infected with viruses in the presence of 4 μg/mL polybrene, and then cultivated in the presence of 250 μg/mL G418, 0.5 μg/mL puromycin, 3 μg/mL blasticidin-S, or 50 μg/mL hygromycin-B for selection. All retrovirus-infected cells were confirmed to be free of helper viruses by horizontal spread assay analysis and western blotting for the viral Gag protein.

### Cell culture

Immortalized cells were cultured in DMEM supplemented with 10% FBS (10437-028, Gibco) and 1´ antibiotic-antimycotic mixed solution (P4333, Sigma-Aldrich) and maintained at 37 °C with 5% CO_2_.

### Population doubling assay

Cells (1 × 10^5^ cells/mL) were seeded in a 25-cm^2^ dish and passaged at 80% confluency. The population doubling level (PDL) was obtained from the number of cells using the following formula: PDL = log_2_(*a*/*b*), where “*a*” is the number of cells counted in the passage and “*b*” represents the number of seeded cells [17].

### Western blot analysis

Cell lysates were prepared by disrupting cell pellets in the Laemmli sample buffer (Bio-Rad, Hercules, CA, USA) supplemented with 5% beta-mercaptoethanol (Sigma-Aldrich), and heated at 96°C for 5 min. Samples were then subjected in duplicate to 4–12% gradient SDS-PAGE (Invitrogen) and transferred onto PVDF membranes using BioRad semi-dry transblotters. Membranes were blocked with LiCor blocking buffer (LiCor, Lincoln, NE) for 1 h at room temperature, and probed with primary antibodies (described in Supplementary Table 1) diluted in LiCor blocking buffer containing 0.1% Tween. The membranes were washed twice for 10 min each in PBS with 0.1% Tween (PBST) and then probed with goat anti-mouse or goat anti-rabbit IR-Dye 670- or 800 cw-labeled secondary antisera diluted in LiCor blocking buffer containing 0.1% Tween and 0.01% SDS for 1 h at room temperature. The probed membranes were washed twice for 10 min in TBST, placed in water, and imaged using a LiCor Odyssey scanner. Boxes were manually placed around each band of interest, and near-infrared fluorescent values for raw intensity, with intra-lane background subtracted, were obtained using Odyssey 3.0 analytical software (LiCor).

### Immunohistochemistry (IHC)

Cells were cultured on Lab-Tek chamber slides (Thermo Fisher Scientific, Waltham, MA, USA) for 24 h and paraffin-embedded; antigens were then retrieved in a sodium citrate buffer. Slides were incubated with primary antibodies (described in Supplementary Table 1) at 41°C and then visualized using a Histofine SAB-PO kit (Nichirei, Tokyo, Japan). When we evaluated the phosphorylation levels of p-erk, p-AKT and p-mTOR, we used appropriate positive controls and negative controls. We performed immunohistochemistry for cell lines below on cell blocks. We used SKOV3 cells as a positive control for p-AKT and p-mTOR and negative control for p-erk. Additionally, we used ES2 cells as a positive control for p-erk and negative control for p-AKT, and MDAH2774 cells as a negative control for p-mTOR. We previously reported that SKOV3 cells are positive for p-AKT and p-mTOR and negative for p-erk, that ES2 cells are negative for p-AKT, and that MDAH2774 cells are negative for p-mTOR [30, 31].

All samples were evaluated by two blinded researchers using a histochemical scoring system (H score), based on the overall staining intensity and the percentage of stained cells, as previously described [4]. For statistical analysis, H scores were classified as negative (> 50) and positive (51 <).

### Fluorescence *in situ* hybridization (FISH)

FISH was performed on 5-μm paraffin sections on a tissue microarray with both tumor and reference cores. ZytoLight^®^ SPEC KRAS/CEN 12 and SPEC PIK3CA/CEN 3 dual-color probes (Zytovision, Bremerhaven, Germany) were used, according to the manufacturer’s protocol. Slides were denatured for 10 min at 75°C and hybridized at 37°C overnight with the probe mix. Cell nuclei were stained with 4′,6-diamidino-2-phenylindole. Signals were evaluated by two independent researchers using an Olympus BX41 fluorescence microscope (Tokyo, Japan). Separate narrow band-pass filters were used to detect the ZygGreen^TM^, ZyRed^TM^, and DAPI signals. Approximately 100 tumor cells were examined for each specimen at 60× magnification, and the number of polysomic cells (≥ 3 signals) was recorded.

### Chromogenic *in situ* hybridization

Before hybridization, samples were deparaffinized in xylene, incubated in 100% ethanol, and then washed with deionized water. The washed slides were placed in an EDTA pre-treatment solution at 95°C for 30 min, and then covered with a pepsin solution and incubated in a humid chamber at 37 °C for 7 min. ZytoDot^®^ *MYC* CISH probe (10 μL) (Zytovision) was added to each sample, and the samples were then denatured in a thermal plate at 94°C for 10 min and transferred to a humid chamber for overnight hybridization at 37°C. The hybridized slides were washed with saline-sodium citrate buffer at 75°C for 5 min and incubated in H_2_O_2_ for 10 min, after which a blocking solution was applied. Next, the slides were sequentially incubated with mouse anti-digoxigenin and anti-mouse-HRP-polymer for 30 min each. For visualization, DAB solution was added for 30 min, and the slides were counterstained with Mayer’s hematoxylin solution, dehydrated, and covered with a coverslip using mounting solution. All steps were performed using the ZytoDot^®^ CISH Implementation Kit Protocol (Zytovision). High c-Myc amplification was defined as large gene copy clusters in > 50% of cells or the presence of > 6 separate gene copies [18].

### Anchorage-independent growth

Cells were resuspended in top agar consisting of DMEM with 5% FBS and 0.33% noble agar, and seeded into a 24-well plate (10,000 cells/well) above a base layer of 0.5% agar in DMEM with 5% FBS. Colonies with diameter > 0.05 mm were counted after incubation for 2 weeks.

### Mouse xenograft experiments

Cells were resuspended in growth medium (2.5 × 10^7^ cells/mL) and subcutaneously or intraperitoneally injected into the base of the left flank of 7–9-week old female BALB/c *nu/nu* mice (Japan SLC, Inc. Hamamatsu, Japan). Tumor growth was monitored weekly for two months or until the mice became moribund. Alternatively, mice grafted with immortalized FTSECs were monitored for 9 months to ensure a non-transformed phenotype.

### Wound healing assays

Cells were seeded in 6-well plates and grown to confluency. An acellular area was created by scraping the cell surface with a 200-μL pipette tip and removing the floating cells by gently washing with culture media twice. The rate of defect closure was monitored for 24 h.

### Matrigel invasion assay

Cell invasion was monitored using chambers with membranes containing 8-μm pores (Discovery Labware, Inc. MA, USA). DMEM containing 30% FBS (750 μL) was added to each lower chamber, whereas approximately 25,000 cells suspended in 250 μL of serum-free medium were seeded in each upper chamber. After culturing for 24 h, the Matrigel was carefully removed using a cotton swab. The membranes were fixed with 4% paraformaldehyde and stained with Giemsa, and the number of migrating cells was counted in five non-overlapping fields using a light microscope.

### Cell proliferation assay

The effect of c-Myc activity on FTSEC proliferation was examined by MTT assay (Sigma) using the c-Myc inhibitor 10058-F4 (Sigma). Briefly, immortalized FTSECs with overexpression of DN-p53, *KRAS*^V12^, and/or c-Myc were seeded in 96-well plates (7,000 cells/well) and treated with 0, 10, 13, 16, 19, or 22 μM 10058-F4 for 24 h. Cell numbers were determined via MTT assays, and data were expressed as a percentage of the DMSO control. Data represent the mean ± standard deviation (SD) from triplicate experiments.

### Patients and tumor samples

Patients with HGSOC (*n* = 34) scheduled to undergo treatment at Shimane University Hospital (Izumo, Japan) between January 2011 and December 2015 were enrolled in our study. Diagnoses were based on conventional morphological examination of hematoxylin and eosin-stained sections. Tumors were categorized based on the World Health Organization subtype criteria [19], and staging and grade were performed according to the International Federation of Gynecology and Obstetrics (FIGO) classification system [20]. All patients were primarily treated with surgery (i.e., total abdominal hysterectomy, bilateral salpingo-oophorectomy, and omentectomy) with or without pelvic lymph and para-aortic node dissection and adjuvant chemotherapy. Half of each tissue sample was examined histologically by clinical pathologists, and the remaining portion was frozen at −80 °C until the mutational analysis. The study protocol was approved by the Ethics Committee of Shimane University (No. 960). All participants provided written informed consent. Research was conducted in accordance with the Declaration of Helsinki and Title 45, U.S. Code of Federal Regulations, Part 46, Protection of Human Subjects, effective December 13, 2001.

### HGSOC mutational analyses

For sequencing analysis, sections from the 34 tumor samples from patients with HGSOC were washed in cold PBS, minced to ~1-mm^3^ fragments, and digested with collagenase A (10 mg/mL) with mild agitation at 37 °C for 40 min. The samples were allowed to rest, and single tumor cells or small tumor cell clusters (< 10 cells) were obtained in suspension after the large undigested tissue fragments settled at the bottom of the centrifuge tube. The tumor cells were washed with PBS, harvested with anti-Ep-CAM (Dynal, Oslo, Norway)-coated magnetic beads, and then subjected to genomic DNA extraction. Samples with limited cell yields were expanded in an RPMI1640 medium with 10% FBS for 3 days, prior to genomic DNA isolation. All exons of *TP53, PTEN*, *KRAS*, and *BRAF*, as well as exons 9 and 20 of *PIK3CA*, were amplified by PCR using the primer sets mentioned in Supplementary Table 2. PCR products were purified using a PCR purification kit (Qiagen, Venlo, Netherlands) and then analyzed by direct sequencing.

## SUPPLEMENTARY MATERIALS FIGURES AND TABLES



## Abbreviations

CA-AKT: constitutively active AKT
EOC: epithelial ovarian cancer
FTSEC: fallopian tube secretory epithelial cells
HGSOC: high-grade serous ovarian carcinoma
OSE: ovarian surface epithelial.
