# Supplementary material for: Reconstitution of high-grade serous ovarian carcinoma from primary fallopian tube secretory epithelial cells
Source: Oncotarget. 2017 Dec 8;9(16):12609–19. doi: 10.18632/oncotarget.23035 (PMC5849158; doi:10.18632/oncotarget.23035)
Supplement: Supplementary file 1 [file oncotarget-09-12609-s001.pdf]

Reconstitution of high-grade serous ovarian carcinoma from primary fallopian tube secretory epithelial cells

SUPPLEMENTARY MATERIALS

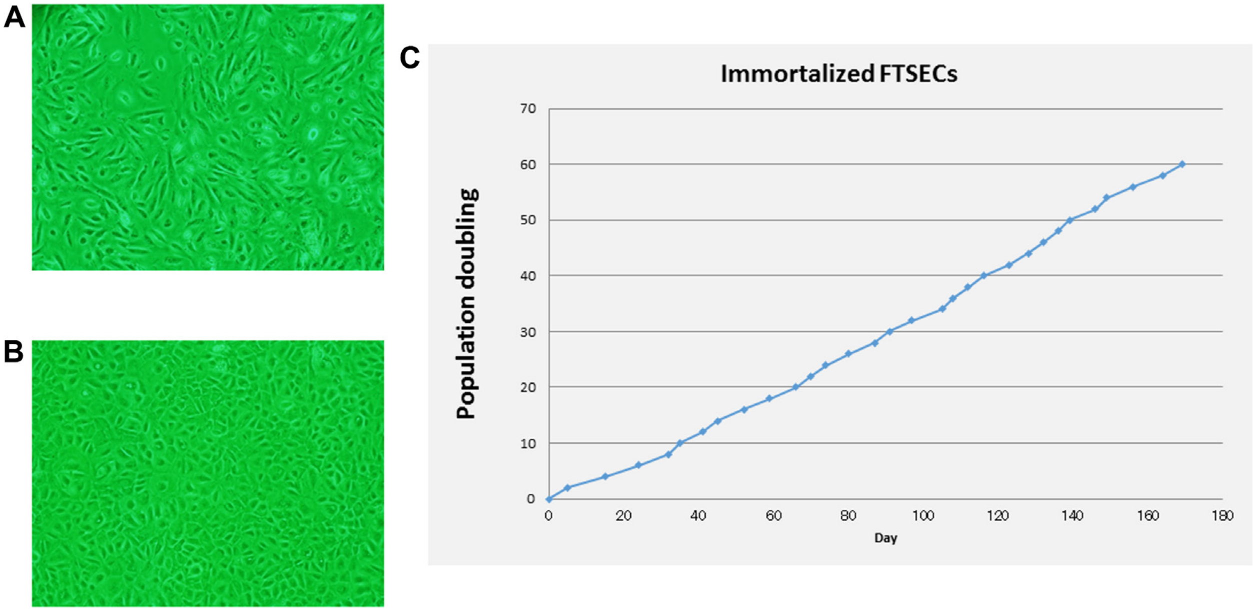

Supplementary Figure 1: Characteristics of primary and immortalized fallopian tube secretory epithelial cells (FTSECs). (A and B) Representative morphology of (**A**) parent primary and (**B**) immortalized FTSECs. (**C**) Growth curve of immortalized FTSECs, as determined via population doubling assays.

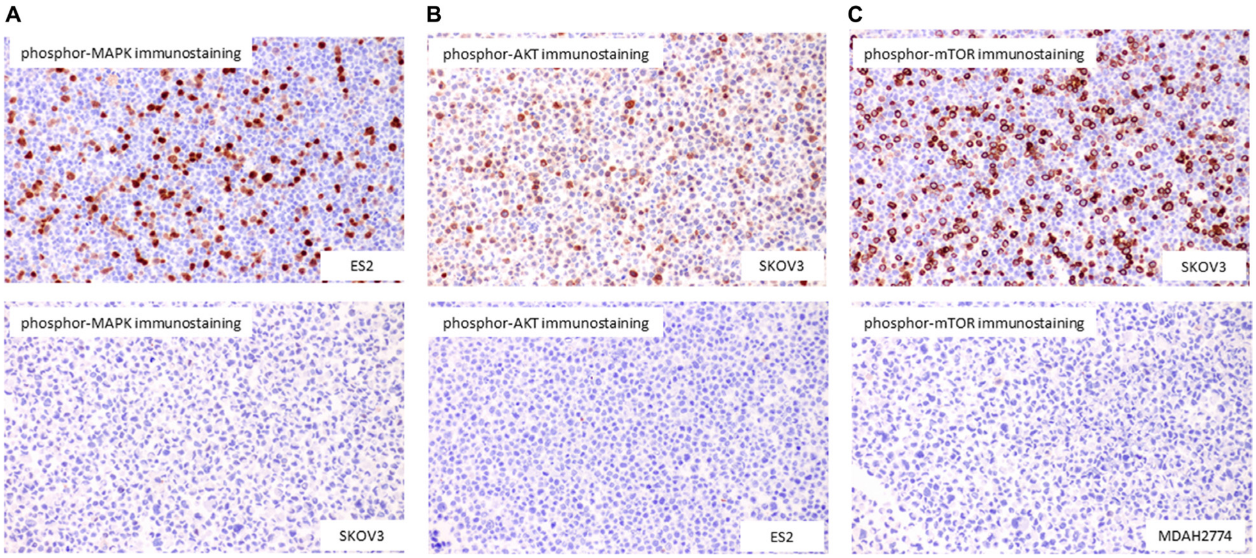

Supplementary Figure 2: Immunohistochemical analysis of phospho-MAPK, phospho-AKT, phospho-mTOR expression in each positive and negative control. Immunohistochemical analysis of (**A**) phosphor-MAPK (ES2: upper panel, SKOV3: lower panel), (**B**) phosphor-AKT (SKOV3: upper panel, ES2: lower panel), and (**C**) phosphor-mTOR (SKOV3: upper panel, MDAH2774: lower panel).

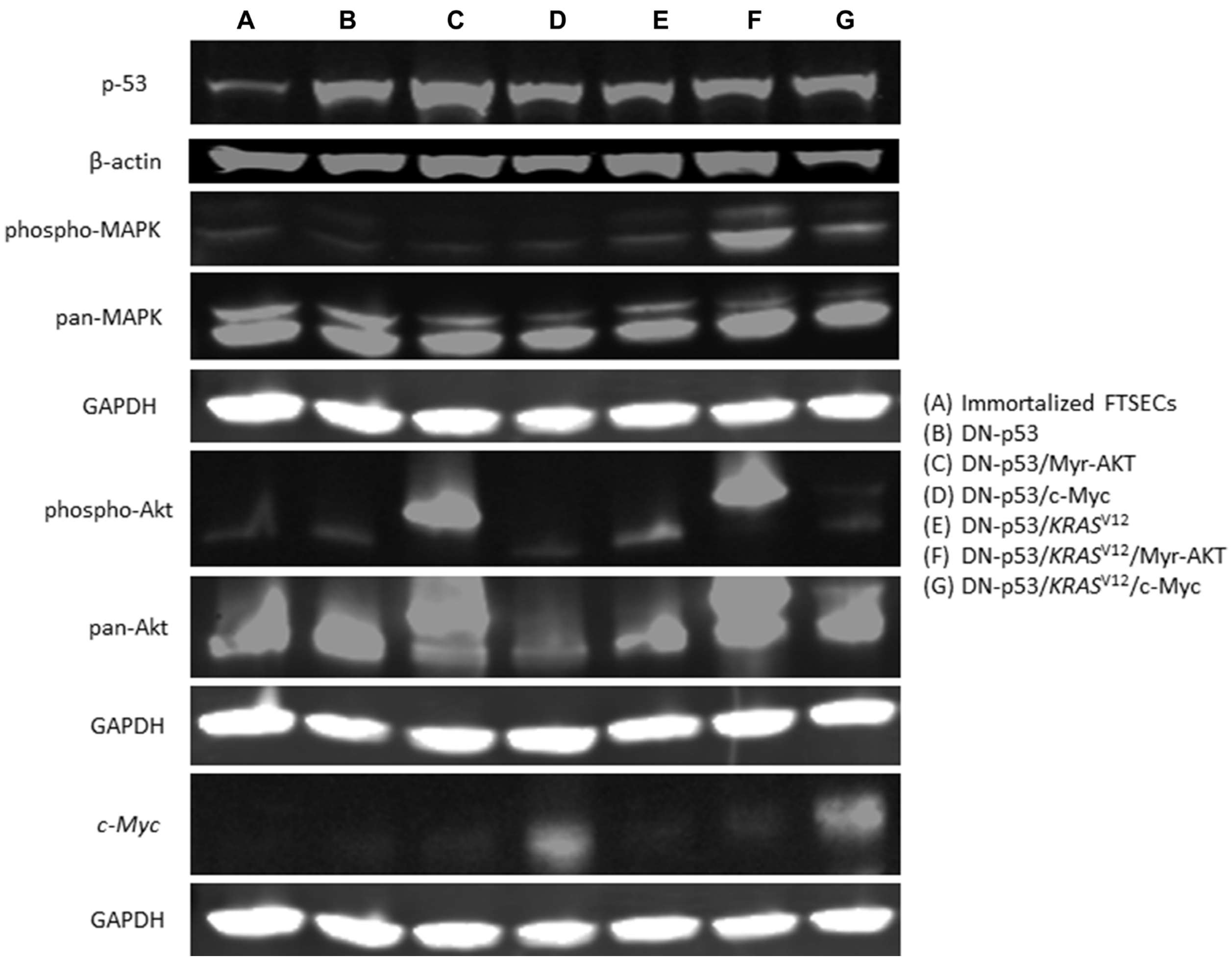

Supplementary Figure 3: Western blot analysis of p-53, phospho-MAPK, pan-MAPK, phospho-AKT, pan-AKT, and c-Myc expression in different transfectants. (**A**) Immortalized fallopian tube secretory epithelial cells (FTSECs), and FTSECs expressing (**B**) DN-p53, (**C**) DN-p53/Myr-AKT, (**D**) DN-p53/c-Myc, (**E**) DN-p53/*KRAS*^{V12}, (**F**) DN-p53/*KRAS*^{V12}/Myr-AKT, and (**G**) DN-p53/*KRAS*^{V12}/c-Myc.

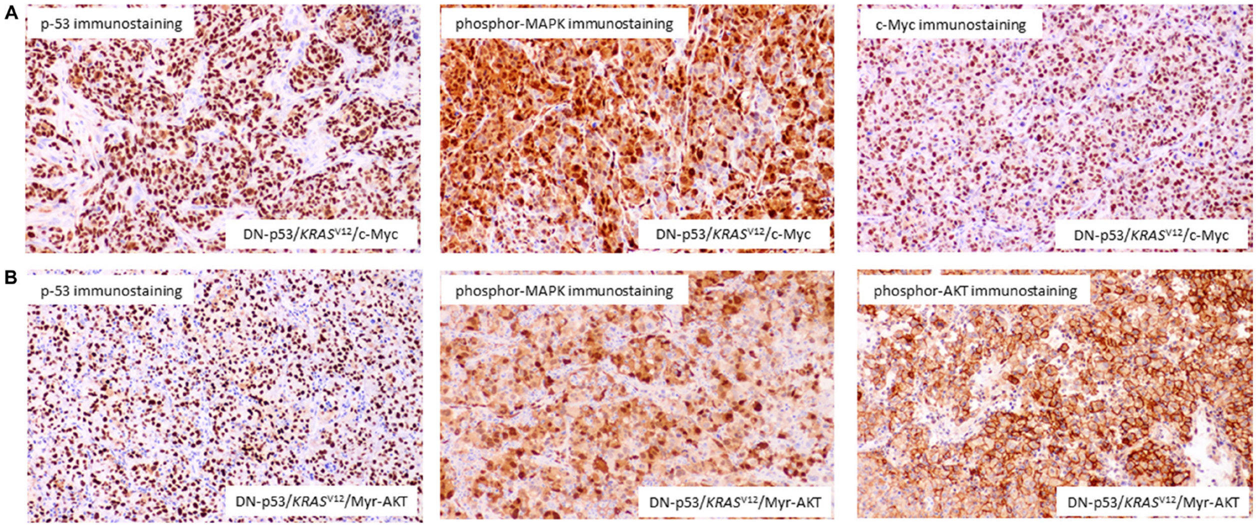

Supplementary Figure 4: Immunohistochemical analysis of p-53, phosphor-MAPK, c-Myc, and phosphor-AKT expression in each xenograft tumor. (**A**) DN-p53/*KRAS*^{V12}/c-Myc (p53: left panel, phosphor-MAPK: middle panel, c-Myc: right panel), (**B**) DN-p53/*KRAS*^{V12}/Myr-AKT (p53: left panel, phosphor-MAPK: middle panel, phosphor-AKT: right panel).

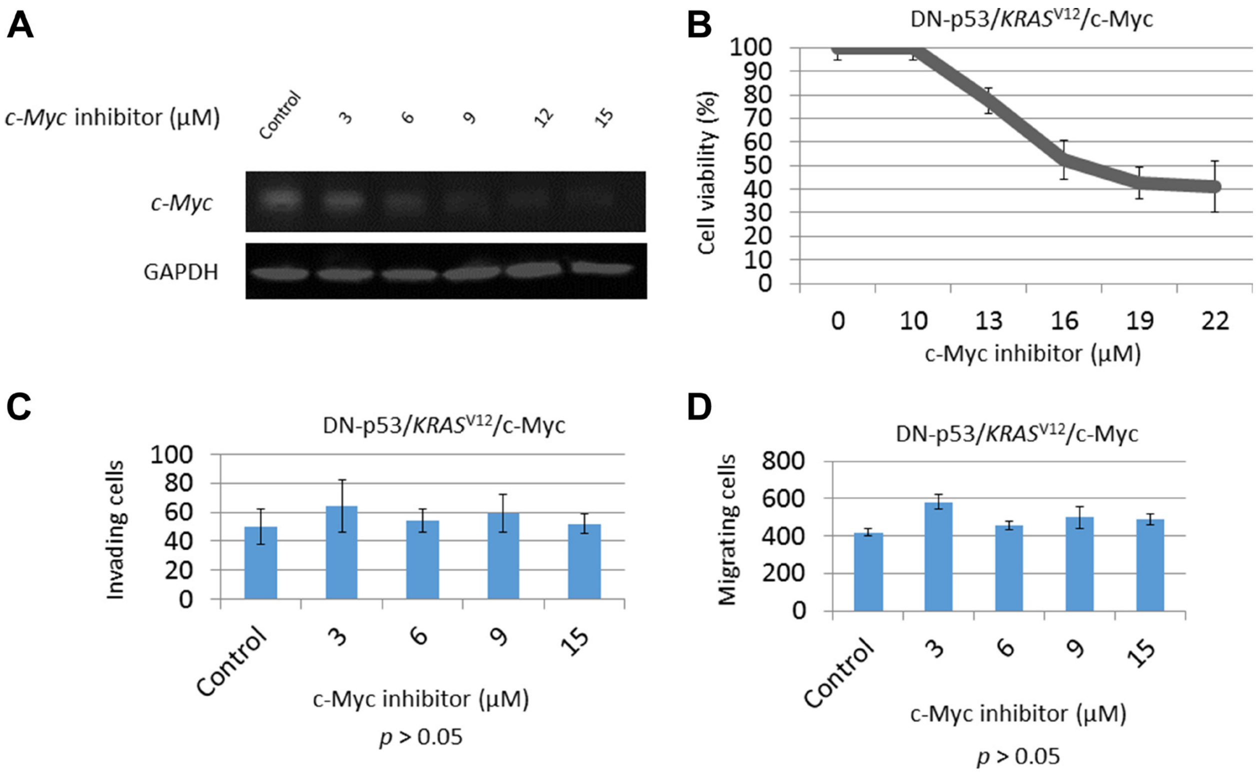

Supplementary Figure 5: Analysis of the effects of a c-Myc inhibitor on the activity of fallopian tube secretory epithelial cells (FTSECs) expressing DN-p53/KRASV12/c-Myc. (**A**) Western blot analyses of c-Myc expression in FTSECs expressing DN-p53/*KRAS*^{V12}/c-Myc upon treatment with different concentrations (3, 6, 9, 12, and 15 µM) of c-Myc inhibitor. (**B**) Viability, (**C**) Matrigel invasion assay analysis, and (**D**) migration of FTSECs expressing DN-p53/*KRAS*^{V12}/c-Myc in a wound healing assay after treatment with the c-Myc inhibitor.

Supplementary Table 1: Description of primary antibodies

Antibody	Manufacturer	Dilution
Anti-pan-Cytokeratin (C11)	Santa Cruz Biotechnology (sc-8018)	1:200 (WB)<br>1:50 (IHC)
Anti-PAX8	Proteintech (10336-1-AP)	1:2,000 (WB)<br>1:1200 (IHC)
Anti-BCL2	Proteintech (12789-1-AP)	1:50 (IHC)
Anti-FOXJ1	Atlas Antibodies (HPA005714)	1:1,000 (IHC)
Anti-WT1	Dako (M3561)	1:100 (IHC)
Anti-CK7	Dako (M7018)	1:100 (IHC)
Anti-CK20	Leica (NCL-L-CK20)	1:50 (IHC)
Anti-p-53	Dako (M7001)	1:50 (IHC)<br>1:100 (WB)
Anti-phospho-p44/42 MAPK (Erk1/2) (Thr202/ Tyr204)	Cell Signaling (#4370)	1:2,000 (WB)<br>1:400 (IHC)
Anti-phospho-AKT (Ser473)	Cell Signaling (#4060)	1:2,000 (WB)<br>1:50 (IHC)
Anti-phospho-mTOR (Ser2448) (49F9)	Cell Signaling (#2976)	1:100 (IHC)
Anti-p44/42 MAPK (Erk1/2)	Cell Signaling (#4695)	1:1,000 (WB)
Anti-AKT (pan) (C67E7)	Cell Signaling (#4691)	1:1,000 (WB)
Anti-c-Myc (Y69)	Abcam (ab32072)	1:10,000 (WB)<br>1:50 (IHC)

WB, western blotting; IHC, immunohistochemistry

Supplementary Table 2: Primers used for PCR amplification and sequencing

KRAS-Exon2-Forward	TTAACCTTATGTGTGACATGTTCTAA
KRAS-Exon2-Reverse	AGAATGGTCCTGCACCAGTAA
BRAF-Exon15-Forward	TGCTTGCTCTGATAGGAAAATG
BRAF-Exon15-Reverse	AGCATCTCAGGGCCAAAAAT
PTEN-Exon1-Forward	TTCCATCCTGCAGAAGAAGC
PTEN-Exon1-Reverse	CAGCCGCAGAAATGGATAC
PTEN-Exon2-Forward	ACTCCAGCTATAGTGGGGAAA
PTEN-Exon2-Reverse	TTTTCTGTGGCTTAGAAATCTTTT
PTEN-Exon3-Forward	TGATTACTACTCTAAACCCATAGAAGG
PTEN-Exon3-Reverse	TTGTTTTAGAAGATATTTGCAAGC
PTEN-Exon4-Forward	AAAGATTCAGGCAATGTTTGTT
PTEN-Exon4-Reverse	TCTCACTCGATAATCTGGATGAC
PTEN-Exon5-Forward	TCCAGTGTTTCTTTTAAATACCTGTT
PTEN-Exon5-Reverse	GATCCAGGAAGAGGAAAGGAA
PTEN-Exon6-Forward	ATATATGTTCTTAAATGGCTACGA
PTEN-Exon6-Reverse	ACATGGAAGGATGAGAATTTC
PTEN-Exon7-Forward	TCATTAAAATCGTTTTTGACAGTTT
PTEN-Exon7-Reverse	TCTGTCCTTATTTTGGATATTTCTC
PTEN-Exon8-Forward	TGTTTAACATAGGTGACAGATTTTCTT
PTEN-Exon8-Reverse	ACAAGTCAACAACCCCCACA
PTEN-Exon9-Forward	TGTTCATCTGCAAAATGGAATAA
PTEN-Exon9-Reverse	CACAATGTCCTATTGCCATTAAA
PIK3CA-Exon9-Forward	GGGAAAAATATGACAAAGAAAGC
PIK3CA-Exon9-Reverse	CTGAGATCAGCCAAATTCAGTT
PIK3CA-Exon20-Forward	CTCAATGATGCTTGGCTCTG
PIK3CA-Exon20-Reverse	TGGAATCCAGAGTGAGCTTTC
P53-Exon1-Forward	CCTTACTTGTCATGGCGACTG
P53-Exon1-Reverse	GAAAATACACGGAGCCGAGA
P53-Exon2/3-Forward	GGGTTGGAAGTGTCTCATGC
P53-Exon2/3-Reverse	AGCCCAACCCTTGTCCTTAC
P53-Exon4-Forward	CCTGGTCCTCTGACTGCTCT
P53-Exon4-Reverse	GCCAGGCATTGAAGTCTCAT
P53-Exon5-Forward	TCAGATAGCGATGGTGAGCA
P53-Exon5-Reverse	CTTAACCCCTCCTCCCAGAG
P53-Exon6-Forward	TCTGTCTCCTTCCTCTTCCTACA
P53-Exon6-Reverse	AACCAGCCCTGTCGTCTCT
P53-Exon7-Forward	CTTGGGCCTGTGTTATCTCC
P53-Exon7-Reverse	GGGTCAGAGGCAAGCAGA
P53-Exon8-Forward	GGGAGTAGATGGAGCCTGGT
P53-Exon8-Reverse	GCTTCTTGTCCTGCTTGCTT
P53-Exon9-Forward	GGAGACCAAGGGTGCAGTTA
P53-Exon9-Reverse	CCCCAATTGCAGGTAAAACA
